# Adult and student perspectives on racial and ethnic equity‐informed school‐based strategies

**DOI:** 10.1002/pits.22575

**Published:** 2021-08-04

**Authors:** Charles H. Lea, Kristin J. McCowan, Tiffany M. Jones, Angela Malorni

**Affiliations:** ^1^ Graduate College of Social Work University of Houston Houston Texas USA; ^2^ School of Social Work University of Missouri‐Columbia Columbia Missouri USA; ^3^ School of Social Work Colorado State University Fort Collins Colorado USA; ^4^ School of Social Work University of Washington Seattle Washington USA

**Keywords:** racial and ethnic equity, students of color, youth development

## Abstract

Although racial and ethnic equity‐informed school‐based strategies are important to addressing racialized structures and processes that create and sustain racial trauma, disadvantage and disparity, little is known about the process of embedding racial and ethnic equity in school‐based strategies and how adults and young people perceive them to promote a positive school climate and youth development. Using a qualitative research approach that included focus groups, this study examined student of color and school and community partner staff perceptions of the role and influence racial and ethnic equity‐informed school‐based strategies in one middle school have on the school's climate and students of color experiences and development. Findings suggest that racial and ethnic equity‐informed social and emotional learning strategies are important in facilitating positive student–teacher interactions and identity and social‐emotional development among students of color. Participant's experiences in and perceptions of the impact these strategies have on school climate and youth development substantiate the need to understand racial and ethnic equity as a process‐oriented approach that requires continuous improvement, rather than just an outcome‐focused endeavor. Implications for research and practice are discussed.

1

In 2016, approximately 1 in 6 (17%) young people aged 6 through 18 in the United States (U.S.) had at least one mental health diagnosis (e.g., anxiety, depression, attention‐deficit/hyperactivity disorder (Whitney & Peterson, 2019). While rates of mental health disorders are similar among young people across racial and ethnic groups, the symptoms of these disorders tend to be more persistent among young people of color (American Psychiatric Association, 2017). Literature points to the structural, institutional, and personal nature of racism and racial discrimination in the United States as critical factors that undermine their mental health because these everyday experiences often lead to racial trauma (Alexander, 2012; Association of Black Psychologists ABPsi & Community Healing Network., 2016; Dixson et al., 2006; Jernigan & Daniel, 2011). Given the racialized structure and climate of schools, they are one context where students of color commonly contend with racism, racial discrimination, and racial trauma (Alexander, 2012; Dixson et al., 2006; Noguera, 2009; Skiba et al., 2011). Developing and implementing of school‐based strategies that embed racial and ethnic equity is identified as an important racial justice approach to promoting positive experiences, development, and outcomes among students of color (Andrews et al., 2019; Ginwright, 2018; Ortega et al., 2016; Paris & Alim, 2014). Yet, existing literature does not offer an in‐depth understanding of the processes that facilitate such embeddedness. More must therefore be done to ensure that the praxis of racial and ethnic equity and justice acknowledges and addresses the ways that racial inequity and injustice in the school context affect the experiences, development, and outcomes of students of color. This paper therefore examines school and community partner staff and student of color perceptions of the role and influence racial and ethnic equity‐informed school‐based strategies in one middle school have on the school climate and student of color's experiences and development.

## 2 RACISM, MENTAL HEALTH, AND THE SCHOOL CONTEXT

According to Thompson (2010), racism occurs in school at the structural, institutional, and interpersonal levels. Racism is embedded in the structure of school because it is a defining feature of American society (Dixson et al., 2006). Racism thus functions as a form of social control in educational systems where its practices and elements promote a culture that allows it to be accepted, normalized, and perpetuated. The historical and current nature of school segregation on the basis of race is just one example of this broad view of structural racism in schools (Ayscue & Orfield, 2016; Kozol, 2005). Moreover, the collaborative and interdependent relationship between educational, political, and economic systems (e.g., residential segregation) help to reinforce and legitimize racism in schools (Reskin, 2000; Thompson, 2010).

School policies and practices that produce disadvantage and disparity among students of color uncover the institutional racism that occurs within educational systems. In particular, many studies document racial and ethnic differences in school suspensions and expulsions, wherein students of color, especially young Black men, are disproportionately pushed out of school and into the juvenile punishment system (Noguera, 2009; Skiba et al., 2011). This phenomenon, referred to as the school to prison pipeline (STPP), undermines these young people's mental health, because the traumatizing nature of this experience can slow their psychosocial development and academic progress (Dmitrieva et al., 2012; Dutil, 2020; Gatti et al., 2009; Grace & Nelson, 2019). School curriculum, which is often based on White middle class values, is another racialized mechanism in schools that impact students of color mental health, because these curricula do not fully honor these students' cultural and linguistic histories and experiences. As such, these young people's racial and ethnic identity development processes are compromised (Dumas, 2016), which can pose challenges and barriers to social and emotional wellbeing (Ginwright, 2018; Kayler‐Jones, 2019). These curricula also promote the ideologies of individualism wherein students of color are often blamed if they perform low on standardized tests, which are racially biased (Fish, 2001).

The racial beliefs and stereotypes that teachers and students have about students of color also become the basis for which personal racism is enacted in the school context (Thompson, 2010). For instance, studies commonly find teachers to have low expectations of their students of color (Andrews & Gutwein, 2017), which is negatively associated with school engagement, academic achievement, and development (Wang et al., 2018). Racial microaggressions, regardless if experienced from a teacher or a peer, also negatively impact students of color schooling experiences and their overall mental health (Nadal et al., 2014). These examples point to the micro, mezzo, and macro ways racism operates in the school context, which suggest a multilevel approach is needed to address the racist structures and processes that are inherent to educational institutions. It is particularly important for these strategies to embed racial and ethnic equity because racist school structures and processes can have lasting effects on a school's climate and culture and student of colors' social and emotional wellbeing (The Aspen Institute: Education and Society Program, 2018).

## 3 RACIAL AND ETHNIC EQUITY IN EDUCATION

Movements, such as Black Lives Matter, have helped to call attention to the need to increase racial and ethnic equity in education as a way to address the racial inequities and injustice students of color commonly experience (Taylor, 2016). According to Andrews et al. (2019), racial and ethnic equity is considered both an outcome and a process:

*As an outcome, racial and ethnic equity is achieved when race and ethnic identity no longer predicts the course of a person's life, and all people have what they need to thrive, no matter where they live. As a process, racial equity is applied when those most impacted by structural racial inequity are meaningful involved in the creation and implementation of the intuitional policies and practices that impact their lives. (p. 5)*.

Although a complex and context‐dependent process (Espinoza, 2007), embedding racial and ethnic equity in school‐based strategies are critical to promoting positive experiences, development, and outcomes among students of color because they create opportunities that help to address the racialized structure and climate of schools that create and perpetuate racial and ethnic trauma, disadvantage, and disparity (Debnam et al., 2014).

Restorative practices, which are rooted in the history and cultures of Black and Indigenous people of color (BIPOC), is one school based strategy that addresses the racial trauma students of color often experience as a result of institutional and personal racism (Sellman et al., 2013). Unlike punitive practices that lead to racial and ethnic disparities in school suspension and expulsion, these strategies seek to proactively build healthy relationships and a sense of community to prevent and address conflict and wrongdoing, which can promote positive individual (e.g., academic, attendance, well‐being) and school‐level (e.g., climate, safety) experiences and outcomes (Ortega et al., 2016). For instance, Ortega et al. (2016) qualitive examination of the influence a Restorative Circle has on student and staff experiences and outcomes found participants to perceive the racial and ethnic equity‐informed strategy to foster opportunities that facilitate positive relationships, academic achievement, and social and emotional wellbeing. Participants also viewed this school‐based strategy as helping to interrupt the STPP.

School curriculum that is culturally and linguistically congruent to the norms, values and experiences of students of color is another racial and ethnic equity‐informed school based strategy. Specifically, this approach to teaching and learning is positively associated with students of color academic, identity and social and emotional development (Skiba et al., 2016). This is especially the case for curricula that is provided by instructors who reflect their cultural and linguistic backgrounds, as racial and ethnic representation helps to create a climate where students of color feel culturally affirmed and safe as they engage in the learning process (Giddings, 2001; Ladson‐Billings, 1995; Paris & Alim, 2014).

Social and emotional learning (SEL), which is characterized by the development of five core competencies (i.e., including self‐ management, self‐awareness, responsible decision‐making, relationship skills and social awareness) is another school‐based strategy that can embed racial and ethnic equity (CASEL, 2020). Specifically, transformative SEL, a process to social and emotional development that acknowledges students' cultural, racial and ethnic, and linguistic backgrounds and experiences, is an emerging racial and ethnic equity‐informed school‐based approach (Jagers et al., 2019). Based on CASEL's core competencies, this justice‐oriented framework integrates students' social and cultural backgrounds through lesson plans and reflection activities and foster collaborative opportunities to identify root causes of inequity and solutions to community and social problems.

### 3.1 Colorblindness in education

Research on the impact of SEL programs have demonstrated decreases in students' involvement in physically aggressive interactions and increases in academic achievement and perceptions of school climate (Catalano et al., 2002; Collie et al., 2011; Espelage et al., 2013; Taylor et al., 2017). However, the effect of SEL programming on students of color is not well understood, as programs rarely report results for specific racial and ethnic groups and some do not include information about race or ethnicity for their sample (Rowe & Trickett, 2018). Moreover, scholars have pointed out that SEL strategies often reflect “White, cisgender, patriarchal norms and values that further enact emotional and psychological violence onto Black, Brown, and LGBTQ+ youth of color” because they are taught to regulate and manage their emotions in ways that do not honor the fullness of who they are (i.e., racial and ethnic identities) (Gregory & Fergus, 2017; Hoffman, 2009; Kayler‐Jones, 2020, p.1). Ginwright (2018) therefore calls for more healing‐centered approaches to youth development that are culturally grounded and views healing as a restoration of identity.

SEL and restorative practices have also been adopted as a universal school‐based strategies, likely because they can offer maximum benefit to school‐wide efforts to address a wide‐range of school‐based challenges (e.g. school climate, bullying, trauma, student misbehavior etc.) (Durlak et al., 2011; 2015; Taylor et al., 2017). While universal approaches are adopted with the goal of benefiting all students, with respect to the social and emotional challenges associated with trauma, there is a growing body of literature indicating that universal approaches fail to acknowledge and address racial and ethnic differences in schooling experiences (Gregory & Fergus, 2017; Hoffman, 2009; Simmons, 2019).

The lack of culturally‐affirming practices embedded in school‐based strategies reflect the colorblind nature of some schools' efforts to address the structures and processes that create and sustain racial trauma, disadvantage, and disparity. Colorblindness emerges from “the belief that racial group membership and race‐based differences should not be taken into account when decisions are made, impressions are formed, and behaviors are enacted” (Apfelbaum et al., 2012, p. 205). The consequences of colorblind approaches to school‐based strategies fail to acknowledge the racialized nature of educational processes and further perpetuates racial and ethnic disparities. Moreover, Simmons (2019) argues that students of color may need to develop a range of skills related to agency and leadership that support their ability to confront injustice, hate and inequity. This suggests that school‐based strategies that address students of color unique experiences in ways that affirm, rather than evade their identities and experiences are critically to promoting racial and ethnic equity and justice in schools (Rolón‐Dow & Davison, 2020; Rowe, 2008; Topor et al., 2018).

## 4 PRESENT STUDY

While school‐based strategies that embed racial and ethnic equity offer a promising approach to addressing the racialized structure and climate of schools and its impact on students of color, existing empirical knowledge does not offer an in‐depth understanding of the processes that facilitate such embeddedness. The present study therefore examined how one middle school embed ideals of racial and ethnic equity within its school‐based strategies and the ways in which school staff, community partners, and students of color experience and perceive these strategies to influence the school's climate and students of color experiences and development. Specific research questions include: (1) How is racial and ethnic equity embedded in school‐based strategies? (2) What influence do these strategies have on school climate and students of color experiences and development?

## 5 METHOD

### 5.1 Research design

This study used qualitative research methods to examine the ways in which school staff, community partners, and students of color in one middle school perceive racial and ethnic equity‐informed school‐based strategies to influence the school's climate and students of color experiences and development (Bhattacharya, 2017; Creswell & Poth, 2018; Merriam, 2009). In particular, a series of focus groups with school and community partner staff and students of color conducted to better understand how racial and ethnic equity is embedded in school‐based strategies, as well as individual and shared attitudes and beliefs concerning the influence these strategies have on students of color experiences and development (Merriam, 2009). With the group processing aspect of focus groups being critical to developing an in‐depth understanding of a topic, social constructivism, which posits that meaning making is co‐constructed, served as the underlying epistemological framework for this study (Berger & Luckmann, 1967; Lock & Strong, 2010).

### 5.2 Positionality statement

With personal beliefs and assumptions and power dynamics and tensions playing an influential role on the research process, we discuss our positionality below (Merriam, 2009). The first author is a cisgender Black man who is a member of the social work faculty at a large university. His research, rooted in his own racialized experiences and practice with students of color, is driven by his belief that educational institutions are inherently racist, and that racial and ethnic equity‐informed strategies can buffer the impact of racial trauma and facilitate positive experiences, development and outcomes among students of color. He led the conceptualization of the study, collected, coded and analyzed the data and led the writing of this manuscript. The second author subscribes to the notion that the existing state of scholarship on the topic of school‐based strategies, namely SEL, lacks criticality, which is, at least in part, due to the fact that critical scholarship is often viewed with disdain or contempt among mainstream youth development scholars. This notion is informed by her lived experience as a Black researcher whose perspectives have previously been misunderstood and often rejected by youth development scholars that uphold the idea that western and positivist paradigms are the “gold standard.” Author two was involved in conceptualizing the study, collecting, coding and analyzing data, and writing this manuscript. While author one and two's positionality situates them as insiders in this study, they acknowledge that their position as university professors make them outsiders, and the fact that they have not served as school staff (Parson, 2019; Stich et al., 2012).

The third and fourth authors identify as White cisgender women at large universities. While both of these authors outsider status as White academics limited their capacity to fully understand the experiences and perspectives of racism in the school, the third author has served as a licensed mental health therapist and the fourth author has facilitated youth participatory action research projects with young people from multicultural and gender diverse backgrounds in school and out of school settings. Both of these authors are motivated by their commitment to undue racism in formal and informal learning spaces. Author three supported the writing of the manuscript and author four supported data collection for one focus group and writing the manuscript.

### 5.3 Study context

A middle school in a large urban district in the Pacific Northwest served as the site for this study. This school, which we refer to as Pacific West Middle School (PWMS), was selected for the study because the authors and district representatives were members of a learning community focused on strengthening the assessment and practice of SEL in school and out‐of‐school settings (Herrenkohl et al., 2020). Through the consortium, university faculty and student researchers were connected with district, school and community‐based organizations (CBOS) to design and support timely research‐practice projects. These local research‐practice projects were linked to priority issues identified by participating members and their organizations, and were facilitated to better equip school districts to monitor and assess their SEL programs for impact, particularly related to addressing race differences in student outcomes. In alignment with the District's “Racial Equity Systems Transformation Plan,” PWMS, which serves 782 students in grades six through eight who primarily identify as a students of color (67%), implemented a range of SEL strategies to facilitate changes in school climate and to address racial inequities that impact student achievement and development. These strategies include adult and student equity teams, antiracist professional development trainings, transformative discipline practices such as healing circles, culturally and linguistically responsive curriculum and SEL programming in collaboration with community based organizations.

Based on the consortium model, this study was developed from conversations with the district's administrator who identified the need to better understand the role and influence the antiracist training and four racial and ethnic equity‐informed programs at PWMS play in creating a positive school climate and supporting students of color development. The antiracist professional development training, which was offered to school and community partner staff, was structured to build individual and collective knowledge and skills surrounding individual and institutional biases perpetuating inequities in instruction, achievement, and discipline, cultural sensitivity with historically marginalized and oppressed groups, and culturally responsive practices with students of color. All school staff and program leaders from Program #2 and #4 in this study participated in the training. Program #1 provides tutoring to young men of African descent and supports them with developing a positive racial identity, social‐emotional awareness, and critical consciousness through an athletic program. Program #2 uses a group, process‐oriented trauma‐informed approach that incorporates mentoring and relationship‐building activities to help young women in seventh and eighth grades adjust to and navigate peer relationships and conflict within the middle school context. Program #3 is a student‐led advocacy program that aims to center youth voices and build skills surrounding teamwork, conflict resolution, and problem solving. In groups, students work collaboratively to design and carry out a school‐wide advocacy project that focuses on some issue of equity. Lastly, Program #4 is trauma‐informed prevention and early intervention that uses a psychoeducation curriculum focused on safety, emotions, loss, and future goals to facilitate empowerment and resilience among students facing traumatic life events.

These school‐based strategies were identified as racial and ethnic equity‐informed because they seek to address issues of race and racism, used restorative practices and curriculum that are culturally and linguistically congruent with students of color, and were facilitated by adults who reflected their backgrounds. While Programs #2, #3, and #4 were offered to students regardless of all racial and ethnic backgrounds, students of color were intentionally targeted and included as part of these programs as they were commonly impacted by the school's racialized structures and processes.

### 5.4 Data collection

Four (*n* = 4) focus groups were conducted at PWMS to illuminate how the racial and ethnic equity processes embedded within school‐based strategies can promote a positive school climate and experiences and development among students of color. Specifically, two focus groups were conducted with nine students of color (see Table 1). The first focus group included six participants (*n *= 6), of which two were enrolled in Program #1 (both cisgender males, one identifying as African American/Black and the other as multiracial/ethnic). The remaining four participants identified as a cisgender African American/Black female and were enrolled in Program #4 (*n* = 2), Program #2 (*n* = 1) and Program #3 (*n* = 1). The second student focus group included three participants (*n* = 3). One identified as cisgender Hispanic/Latinx male and was enrolled in Program #3, the second identified as a cisgender African American/Black male and was simultaneously enrolled in Program #3 and Program #1, and the third identified as a cisgender multiracial female who was enrolled in Program #2. A third focus group was conducted with eight (*n* = 8) school staff (administrators, teachers, instructional coaches, restorative justice coordinators), of which 50% identified as a BIPOC, and the fourth was conducted with three staff from the CBOS partnering with PWMS who all identified as a BIPOC (see Table 2).

**Table 1:** Characteristics of student focus group participants

	Frequency	Percent
Gender		
Cisgender male	4	44%
Cisgender female	5	56%
Race/ethnicity		
African American/Black	6	67%
Hispanic/Latinx	1	11%
Multiracial/ethnic	2	22%
Age		
13	4	44%
14	5	56%
SEL Program^a^		
Program #1	3	30%
Program #2	2	30%
Program #3	3	20%
Program #4	2	20%
Length of Time in SEL Program		
Less than 12 months	2	20%
12 > 24 months	4	40%
Missing data	4	40%

^a^ One young person was simultaneously enrolled in Program #1 and Program #3.

**Table 2:** Characteristics of the school staff focus group participants

	Frequency	Percent
Gender		
Cisgender male	4	50%
Cisgender female	4	50%
Race/ethnicity		
African American/Black	1	12.5%
White	4	50%
Native Hawaiian/Pacific Islander	2	25%
Multiracial/ethnic	1	12.5%
Age		
21–30	2	25%
31–40	3	37.5%
41–50	2	25%
50+	1	12.5%
Years at Pacific West Middle School		
1–2	4	50%
3–5	3	37.5%
6+	1	25%
Teaching certificate		
Yes	7	87.5%
No	1	12.5%
Subject area		
Special education	2	25%
Mathematics	1	12.5%
Social studies, history, geography	3	37.5%
English language arts	3	37.5%
Other	4	50%
Highest degree		
GED	1	12.5%
Bachelors	2	25%
Masters	4	50%
Doctorate	1	12.5%

To identify and recruit potential school and community partner staff, we worked closely with the District representative and PWMS's principal. Specifically, the district representative facilitated an email introduction to the principal, who we then worked with to identify school leaders, teachers, and community partners participating in or facilitating racial and ethnic equity informed school‐based strategies. To recruit students of color, school and community partner staff were asked to share a study flyer and consent form with the parents of students participating in their racial and ethnic equity‐informed SEL programming. Students who returned a signed parental consent were able to participate in a focus group discussion. Given the district, school, and community partner staff involvement in recruitment, a parent consent rate is unavailable.

A semi‐structured interview protocol that explored participants' experiences and perceptions of PWMS's racial and ethnic equity‐informed SEL strategies on the experiences and development of students of color was used to facilitate each group discussion (see Table 3). Each focus group lasted approximately 1 h, was conducted in a private room at the school, and audio recorded with participants' permission. Light refreshments were provided at each focus group session, and the students also received a $10 gift card for participating in the group discussion. A gift card was not provided to adult participants due to a district policy. In advance of the study's start date, human subjects' approval was provided by a university‐level Institutional Review Board.

**Table 3:** Questions by focus group type

Focus group	Sample questions
Student	Can you tell me about your experience at the school? Program?
	How do the staff make the program content and activities relevant to your life?
	What impact has this program had on you (identity, emotions, relationships)? Your school?
School staff	How is the school and classrooms structured to support students of color academic and social‐emotional development?
	How would you describe your experience participating in the antiracist professional development training?
	What have you started doing differently in your teaching/practice after participating in the training? What impact has this had on your classroom climate? What challenges have you faced implementing changes?
Community‐based organization	Can you describe the structure of your program, including the content discussed and related activities?
	How do you ensure that program content and activities are relevant to the culture and experiences of students of color?
	What impact has your program had on students of color identity, academic, and social emotional development? School and classroom climate?
	What improvements or changes are necessary to better support your programming efforts.

### 5.5 Analysis

A thematic analysis approach was used to facilitate data analysis and interpretation (Braun & Clarke, 2006; Creswell & Poth, 2016). First, each professionally transcribed focus group session was added to Dedoose, a web‐based software program for qualitative data analysis, and then reviewed for accuracy and to identify emerging codes. Emerging and a priori codes derived from the research questions and interview protocols were then identified and defined to develop an initial codebook. All data was then coded using the codebook, in which codes and the coding process was refined based on researcher discussions. Individual codes were then combined into conceptual categories, and categories were then defined and compared and contrasted to identify emerging themes. The research team also wrote analytic memos to discuss emerging codes, categories, and themes, as well as to be reflective of the biases and assumptions influencing the analytic process (Charmaz, 2006). Rigor and trustworthiness strategies employed in this strategy include peer debriefing, memoing, and triangulation (Forero et al., 2018).

## 6 RESULTS

The findings suggest that participants perceived PWMS' school‐based SEL strategies to embed racial and ethnic equity when they acknowledge and address the influence racism has on students of color schooling experiences and outcomes and center adult and young people's racial identity, culture, and lived experiences. Incorporating this content and activities in the SEL strategies was perceived to broaden some of the adult's understanding of the structural and systemic origins of racial inequity and inequality, which in turn shifted their practices. They also perceived this content and activities to facilitate positive schooling experiences and healthy development among students of color. Two themes, (1) acknowledging and addressing the influence of racism and (2) centering race, culture, and lived experience, help to describe these findings in greater detail.

### 6.1 Acknowledging and addressing racism

#### 6.1.1 Teaching and learning

Engaging school and community partner staff in antiracist professional development training is one strategy PWMS employed that helped them acknowledge and address the influence racism has on students of color schooling experiences and outcomes. In particular, participants in the school and community partner focus group perceived the training to enhance their understanding of the historical nature of structural and institutional racism that exists beyond PWMS. This helped to foster a collective commitment and recognition that ongoing conversations about racial and ethnic equity are ubiquitous and necessary to address racially inequitable policies and practices at PWMS. One participant expressed:

*My takeaway is that the issues are systemic. They go above Pacific Middle, above [this district and community]. They're ingrained in the history that we have in the United States, and it seems insurmountable to take that on. But we have shown in some of the policies that we have implemented or taken away that it is possible to make change*.

This was echoed by another participant in the school staff focus group who expressed, “I think coming together in that training was very important for us to put our minds together and work collectively.” As a result, participants described how they shifted their practices to ensure they created equitable schooling experiences and opportunities for students of color.

One strategy PWMS employed to address the racialized structures and processes inherent to its academic approach was implementing the Cambridge Model in all classrooms. This approach to teaching and learning uses an international Baccalaureate education model that was described by participants in the school staff focus group as “fast‐paced,” “rigorous,” and “having higher expectations than non‐Cambridge classrooms.” While participants perceived the Cambridge classrooms to be more academically rigorous, in terms of preparing students for secondary and postsecondary education, students of color, with the exception of Asian American students, were disproportionately represented in non‐Cambridge classrooms. One participant in the school staff focus group explained:

*We used to be a divided community where half the school was Cambridge and half the school wasn't. When you looked at the classrooms, oftentimes you would see Asian and White students in Cambridge and Black and Brown students in non‐Cambridge classes*.

While the decision to adopt the Cambridge Model occurred before participants' engagement in the antiracist training, the training, as one participant explained, “validated the risky move of making the school all Cambridge.”

Participants involved in teaching and learning at PWMS perceived their engagement in the training to help center dialogues about antiracist pedagogy at the school that were not occurring before the training. By centering antiracist pedagogical practices, they were able to identify and address the ways the school's policies and practices, especially related to teaching and learning, reflected a culture of individualism rather than collectivism. For instance, one participant explained:

*Prior to that training, I think [how we approached teaching and learning] was very individualistic. I had my own ideas; other teachers had their own ideas…. It had been a while since I had learned that stuff in graduate school. So, it kind of re‐centered me into that anti‐racist paradigm so that was helpful*.

Some participants, therefore, expressed being more intentional and using critical reflection in their approach to teaching and learning to ensure they provided opportunities for students of color to feel safe and represented throughout the learning process. One participant explained:

*When I create a lesson plan or when I deliver a lesson, I'm thinking about whether that would be something that would trigger someone or whether that might isolate certain groups…. I'm way more focused now. We have a standard that's about understanding history from a variety of perspectives. I think that's the standard I've used most this year. Before [the training], it was always, check off all the boxes … and now it's more like we have to understand all of the perspectives*.

Yet, while the antiracist training promoted more critical reflection with regards to teaching and learning, some participants expressed facing barriers and challenges with ensuring they captured the diversity of their students of color given the limitations of the required curriculum. For instance, one participant expressed:

*I think the curriculum, at least at the sixth grade [level], is so diverse because they're using a Chinese narrator…. But it's like trying to force these little pieces of diversity because it's not a true reflection of the students who are in front of us. So, there's very little wiggle room in terms of the texts…. [so,] that's something that I'm struggling with*.

Along these lines, while participants in the school staff and community partner focus groups perceived their approach to teaching and learning to be relationship‐based before the training, the training itself promoted them to be more courageous and intentional. In particular, participants expressed feeling less fearful to facilitate difficult conversations with students surrounding issues of race and racism following the antiracist training. Participants in the student focus group recognized this shift, wherein some expressed that they've learned “not to judge someone just because they're a different race. To accept anyone into the group or into anything no matter … what color they are.” Other student participants pointed to discussions about race and racism as affording them the opportunity to help out other students, particularly when someone is “experiencing the problems [associated] with [race].” Participants in the school and community partner staff focus groups also perceived these conversations to help students of color feel validated, especially when they “hear a White person say, ‘yes, I believe you and I trust you, and this is your experience. They get that message from other people of color in their life but not necessarily White folks.” Yet, although the participant's it the school and community partner staff focus groups viewed themselves as facilitators of these conversations, some perceived the training to help them understand that students of color are experts in their lived experiences and that adults must “be willing to hear something [they] may have never heard before and go, what does that mean? And expect that [the students are] going to give [them] as much of an education as [they're] hoping to provide them.”

#### 6.1.2 Discipline policies and practices

In addition to teaching and learning, participants also shifted PWMS's uniform policy and practices due to racially disproportionate enforcement. According to one participant in the school staff focus group, “The students that were gaining violations for their dress were oftentimes students of color. And I was sick and tired of watching them be pulled out of class to change and then miss academic learning time.” Yet, following the antiracist training, efforts were made to eliminate the school uniform policy, and school staff expressed being intentional about keeping students in the classroom. This shift was viewed as racially and ethnically equitable because it ultimately, “forces [school staff] to have to build a relationship with [students of color] and figure out the best way to help them learn.”

However, one teacher who identified as White disagreed with shifting the uniform policy and perceived the change as lacking structure, consistency, destabilizing the school's climate, and stimulating divisiveness among teachers. They explained:

*I would say that some of our culture here at the school … has taken a step back… We don't have the dress code policy, but we still are supposed to have some type of policy, but we don't uphold that policy. The do‐rags are a big issue that they hadn't been before. I thought we had the policy that you weren't supposed to wear them, but now there's like, it just seems like whatever our policy is now there's exceptions to that policy. I see a lot of that happening. I think some of that causes [the] staff divide a little bit*.

Recognizing that this participant alluded to students of color in mentioning “do‐rags”, another participant in the school staff focus group pointed to the limitations of the antiracist training related to knowledge and skills in facilitating difficult conversations with adults surrounding issues of race and racism. They explained:

*I didn't leave [the training] understanding how [to] have conversations with people that don't agree with us because discipline is a huge thing that teachers don't agree on…. So, I don't know how to have that conversation outside this room. I know we need to do it, but how do you bring people in…Not being critical, but I think that's the next sort of step that we didn't really get to*.

Nevertheless, in terms of the impact shifting the school and classroom policies and practices had on their interactions with students, participants in the school and community partner staff focus groups perceived the antiracist training as valuable in helping them build authentic relationships with their students of color.

### 6.2 Centering race, culture, and lived experience

#### 6.2.1 Conversations about race and culture

Facilitating explicit conversations about race and culture was an essential part of the school‐based strategies facilitated by PWMS's community‐based partners. According to one participant in the community partner focus group, “We talk a lot about culture. We have youth from all over. It's a global community in our tiny group, so it feels like an essential element to the success of our group.” Another participant explained, “We like to talk about deep things … [like] racism and institutional racism and micro and macro aggressions.” Participants in the school community partner staff focus groups also intentionally structured their classrooms and programs in ways to help students of color affirm the positive aspects of their racial and ethnic identity. For instance, one participant explained, “first thing we do is go through our cadence and recite … ‘I am king. I am king. I am royalty…. We've got a whole list.”

Conversations about race and culture were especially common in Program #1 because the underlying organizational mission “[is about] being Black men… supporting other Black men and Black women.” The explicit centrality of Blackness and Black male identity was weaved throughout every aspect of this program, including the overall goals, the focus of various activities, and the content of conversations that center on race and culture. For instance, the CBO focus group participant from Program #1 stated that they facilitated an activity with their Black students about family lineage. A key goal of this activity was to illustrate, for the young people, the difference between African American's awareness of their familial history compared to those from families who have recently migrated to America. As they explained:

*I do that to expose the fact that [African Americans] don't know … what country we really originated from. So, that really… opened the door to [explain to African American students] the reason why that happened…. Our history has been stolen from us, and this has been something that has been going on for over five hundred years. So, you have to understand that that's why you don't know what country you come from*.

This participant perceived that incorporating this activity and content helped their young people understand the valuable nature of becoming more knowledgeable about their familial history because it can support the development of a positive racial identity. Participants in the student focus group also perceived the school‐based strategies that included activities and content that centered race and culture to support their personal identity development and how they viewed others who are different than them. For instance, one student participant who was enrolled in Program #2 expressed:

*[We read this] book and it was about loving yourself as different races and different looks, like hair puffs and different aspects of you, and that you are you. Nobody's opinion matters but yours. I feel that [book] helped me love myself more and understand that not everyone's gonna understand you but you…. [It] really helped me change as a person and the way I look at things and the people*.

Exposing students of color to professionals who share their racial and ethnic background was another way in which race and culture were centered in PWMS's strategies. Participants in the community partner focus group saw this as important to the young people's development because, as one participant put it, “letting them see Black people in a positive light is something that they don't see very often, or talking to them or exposing them to different opportunities.” Black students in Program #1, for instance, was therefore afforded the opportunity to attend a field trip to a neighboring city where they met Black professionals in corporate and athletic careers. As part of this trip, they were able to tour the corporate facility and dialogue with the professional about their career paths. They also visited a local university where they met college students that were members of the Black Student Union. The community partner focus group participant from Program #1 shared that the students were able to “hang out and talk about the Black Student Union and talk about their careers and their goals.” This participant thus perceived that exposing their young people to Black adults in a variety of settings helped to affirm their racial and ethnic identity and support their future goals and aspirations.

#### 6.2.2 Centering lived experience

In addition to exposing students of color to professionals who share their racial and ethnic background, centering adults and young people's lived experiences as part of the learning process was perceived to embed racial and ethnic equity and support students of color development. In particular, participants in the student focus group described Program's #1, #2, and #4 as a place where they can talk about anything on their mind, share their emotions, and receive advice so that they can resolve the issues they are facing. Talking circles, which are rooted in Indigenous values of sharing, respect, and honor, were one of the strategies used to center lived experiences through the SEL process. Participants in the student focus groups who participated in talking circles expressed that they were often instructed to “speak from the heart [and] listen from the soul.” This structure and culture helped many young people feel safe and comfortable when sharing in their groups. One participant in a student focus group described their experience entering their group, and the specific aspect that cultivated an emotionally supportive environment: “When you go into the group it's a real comfortable, supportive environment really. If you have anything on your mind or something's going on with you, you could talk about it there. You feel safe.”

The ability of program advisors and other young people to relate to the lived experiences of participants in the student focus groups was a key aspect of the program environment that they perceived to support students of color with feeling safe and comfortable with expressing their emotions. For instance, in describing their approach to facilitating group discussions, one participant in the community partner focus group explained:

*Our goal is to give them space to show those emotions so we can talk about our experiences of growing up and how we held emotions in and were told, “you better not cry. You aint no punk…. We talk a lot about fatherlessness and fatherhood, and things like that…. It's a lot of tears you know. It gets pretty deep. And then us being Black men in that space … we're trying to show them the dynamic of how Black men can lead a space and be leaders in a space, then show them an example of positivity and then also be there emotionally for them*.

This approach resonated with a participant from a student focus group who enrolled in this program, as he viewed their shared lived experiences as important to his experiences and development. He explained, “[the program advisor] created the group because he wanted to help [and] used to be in that position…. So, if you are struggling [in life], he will sit you down and talk to you … I feel like it's just what I needed, support.” The community partner staff member from Program #1described how students with similar lived experiences also supported one another by empathizing with each other during their group sessions. He explained, “this kid's father died, you know I guess one of the students came up to him and supported him and then he decided to join the group and he's with us.”

Centering lived experiences as part of the school‐based SEL strategies was also perceived to support young people with identifying and expressing difficult emotions in a healthy way. For instance, a participant in the community partner focus group explained:

*[During group] yesterday, a young person was sharing that her 16 year old brother at one of the local high schools had been beaten unconscious in relation to a shooting that took place in our district earlier in the year…. And this young person said, ‘there's no place for me to have to have been able to drop that off and peace that out.’*

Participants in the student focus group echoed the notion that centering lived experiences supported their social emotional development, especially related to interpersonal and leadership skills, conflict resolution, future orientation and community building. For instance, one participant in the student focus group expressed:

*Sometimes there's drama between someone and another person, so we just talk it out and see the different perspective and stuff, and how we could solve those problems. And then sometimes we talk about different problems around the school and how we can be leaders of other people to be better*.

A second student participant expressed:

*[This program] is to get you off the streets instead of doing drugs or whatever, or keeping you out of fights and stuff. It helps kids realize that they don't need to do that stuff. There's always another way in life. And sometimes I'll go to church with them and they have breakfast in the morning and talk about our life and stuff. Well help each other out. Participant 2*.

Other students also perceived centering lived experience to have an overall positive impact on the school climate as a whole:

*[Program #3] helps make the school a better place and feel more comfortable with kids…. And it's really open. Whatever is on our mind we can just say it. If there's an issue, something that we notice, we can go there and we'll get that problem resolved*.

## 7 DISCUSSION

This study examined how school and community partner staff and students of color in one middle school perceive strategies that embed racial and ethnic equity to influence the school's climate and students of color experiences and development. Findings suggest that racial and ethnic equity‐informed strategies are important to facilitating positive student–teacher interactions and identity and social‐emotional development. Participants also situated the racial and ethnic equity‐informed strategies in a broader context, in which they identify these initial strategies as the beginning of a longer term transformation of the school. Their overall experiences and perceptions of the impact these strategies on school climate and youth development substantiate the need to understand racial and ethnic equity as a process oriented approach that requires continuous improvement, rather than just an outcome‐focused endeavor.

In line with existing literature, study findings demonstrate that a commitment to acknowledging and addressing racism throughout the learning process plays a critical role in embedding racial and ethnic equity (Collie et al., 2011; Skiba et al., 2016). The antiracist training, in particular, prompted this commitment because it helped to create a shared understanding of the structural and systemic ways in which racial inequity occurs throughout the learning process. School and community partner staff who participated in this training therefore expressed being more intentional and courageous about centering antiracist pedagogy, as well as using restorative practices to redress racial disparities in school discipline. These steps were viewed as supporting a positive school climate and youth development because it created equitable opportunities for students of color to learn, express their emotions, and develop skills in a space that honors their strengths and social and cultural histories (Giddings, 2001; Ladson‐Billings, 1995; Ortega et al., 2016; Sellman et al., 2013).

Similar to the literature on healing‐centered engagement (Ginwright, 2018; Kayler‐Jones, 2019; Ladson‐Billings, 1995; Paris & Alim, 2014), participants' perceptions of the racial and ethnic equity‐informed strategies illustrate that building trust is a prerequisite that subsequently creates an emotionally safe environment that promotes youth development. Across all focus groups, trust‐building led to emotional comfortability that laid the foundation for students of color to talk openly about their experiences with trauma, or otherwise. While building trusting relationships between and among adults and young people can be challenging in a general sense, talking circles or structured check‐ins were an important strategy that all the school‐based strategies used to facilitate a positive climate and relationship building. Discussions of race and racism were also aspects of the school‐based strategies that helped to promote positive relationships and identity development. These findings support the idea that trauma is a collective experience and that strategies that seek to address it must view healing as a restoration of identity that involves community building.

The findings also indicate that including content and activities related to race and culture is essential to students of color development of a positive racial and ethnic identity. Yet, including content and activities that reflect students of color race and culture is only one part of embedding racial and ethnic equity. The school‐based strategies in this study that were facilitated by adults who reflected the racial and ethnic background and experiences of students of color, such as Program #1, appeared to more deeply embed racial and ethnic equity throughout the learning process (e.g., family lineage activities, exposure to Black professionals, etc.). This finding illuminates the important role and influence racial and ethnic representation plays in embedding racial and ethnic equity in school‐based strategies, because students in this program expressed feeling safe and supported, which helped to facilitate positive identity, social emotional development, and future orientation (Giddings, 2011; Ladson‐Billings, 1995; Paris & Alim, 2014).

### 7.1 Implications for embedding equity into school mental health

Given the complex and context‐dependent process of embedding racial and ethnic equity in schools (Espinoza, 2007), it was not surprising that participants involved in teaching and learning perceived the curriculum as limiting given the diverse racial and ethnic backgrounds of their students. Additionally, other participants did not see the inherent value of using restorative practices to address student misconduct. While it is likely that these participants attribute racial disparities in school discipline to student's behaviors rather than racial bias (Gregory & Fergus, 2017; Hoffman, 2009; Simmons, 2019), the findings point to the importance of engaging school and community partner staff in ongoing professional development activities that help them to examine and address how their own privilege, power, and biases show up throughout the learning process. This type of self‐awareness lays the foundation for them to better understand how their ideologies, beliefs, and actions support or hinder racial and ethnic equity processes in education and students of color experiences and outcomes.

Implementing racial and ethnic equity‐informed strategies in education also requires an investigation of the implicit culture within the school. Without a framework that maintains conscious awareness of racial and ethnic and social differences it becomes increasingly challenging to see how racial and ethnic equity‐informed strategies may further perpetuate racial disparities in educational and mental health outcomes (Apfelbaum et al., 2012). Using intersectionality as a guiding framework is one way that researchers, policymakers and practitioners can begin to uncover the multiple ways in which racialized school structures and processes intersect with other aspects of the students of color identities (gender, class, sexuality, gender, etc.) (Crenshaw, 1991). One way to do this is to ensure that school and community partner staff implementing these strategies have similar identities and experiences as the students of color within the school context, because it is critical for them to feel safe in school and explore their racial identity and express racialized experiences in healthy ways. Partnerships with CBOSs are essential to embedding racial and ethnic equity in school‐based strategies, because these organizations tend to have more flexibility in their approach compared to school staff, namely teachers, who are bounded by district and state mandated curriculum.

## 8 LIMITATIONS

The study includes some limitations. First, because focus groups served as the primary approach to data collection, interviews or observations could have strengthened the study, allowing for more triangulation across data sources. However, because multiple stakeholders from the school site were involved (i.e., students and school and community staff), we were able to gain multiple perspectives about the ways in which racial equity is embedded in SEL strategies and their impact on students of color. The use of a convenience sampling approach was another limitation of this study, as the study population does not reflect those of all members of school.

## 9 CONCLUSION

Through a series of focus groups with school community partner staff and students of color, this study examined how racial and ethnic equity‐informed school‐based strategies influence school climate and the experiences and development of students of color. Results indicate that racial and ethnic equity strategies are important to facilitating positive student–teacher interactions and identity and social‐emotional development among students of color. Participant's perceptions of the impact of these strategies on school climate and youth development substantiate the need to understand racial and ethnic equity as a process oriented approach that requires continuous improvement, rather than just an outcome‐focused endeavor. By embedding ideals of racial and ethnic equity, schools have a unique opportunity to create opportunities to address the colorblind notion of existing strategies that seek to dismantle the racialized structures and process that lead to racial trauma, disadvantage, and disparity. Race‐conscious school‐based strategies can also enhance opportunities for students to process their racialized experiences, develop skills and to address the social and emotional impacts of racism. Future studies that examine the extent to which embedding racial and ethnic equity in school‐based strategies has the opportunity to address structural racism are needed.

## 10 PRACTITIONER POINTS

- Partnerships among school and CBOSs are essential to embedding racial and ethnic equity into school mental health.
- SEL facilitators with shared racial and ethnic identities and lived experiences are important to promoting social and emotional development among students of color.
- Ongoing support surrounding antiracism is critical to promoting SEL facilitator's identity and social‐emotional development and commitment to racial and ethnic equity‐informed practice.

## CONFLICT OF INTERESTS

The authors declare that there are no conflict of interests.

## References

[pits22575-bib-0001] Alexander, M. (2012). The new Jim Crow: Mass incarceration in the age of colorblindness. The New Press.

[pits22575-bib-0002] American Psychiatric Association . (2017). *Mental health disparities: Diverse populations*. //Volumes/GoogleDrive/My%20Drive/1.%20Research%20%26%20Scholarship/2.%20Projects/SEL%20Consortium%20V2/SEL%20Consortium/Research/Papers/Embedding%20Racial%20Equity/Literature%20/Mental-Health-Facts-for-Diverse-Populations%20(1).pdf

[pits22575-bib-0003] Andrews, D. J. C. , & Gutwein, M. (2017). “Maybe that concept is still with us”: Adolescents' racialized and classed perceptions of teachers' expectations. Multicultural Perspectives, 19(1), 5–15. 10.1080/15210960.2016.1263960

[pits22575-bib-0004] Andrews, K. , Parekh, J. , & Peckoo, S. (2019). *How to embed a racial and ethnic equity perspective in research: Practical guidance for the research process*. Child Trends. https://www.childtrends.org/wp-content/uploads/2019/09/RacialEthnicEquityPerspective_ChildTrends_October2019.pdf

[pits22575-bib-0005] Apfelbaum, E. P. , Norton, M. I. , & Sommers, S. R. (2012). Racial color blindness: Emergence, practice, and implications. Current Directions in Psychological Science: A Journal of the American Psychological Society, 21(3), 205–209. 10.1177/0963721411434980

[pits22575-bib-0006] Association of Black Psychologists (ABPsi) & Community Healing Network . (2016). *Family community self‐care toolkit: Healing in the face of cultural trauma*. https://www.abpsi.org/pdf/FamilyCommunitySelfCareToolKit.pdf

[pits22575-bib-0007] Ayscue, J. B. , & Orfield, G. (2016). Perpetuating separate and unequal worlds of educational opportunity through district lines: School segregation by race and poverty. In P. A. Noguera, J. C. Pierce, & R. Ahram (Eds.), Race, equity, and education: Sixty years from brown (pp. 45–74). New York, NY: Springer.

[pits22575-bib-0009] Berger, P. L. , & Luckmann, T. (1967). The social construction of reality. Doubleday.

[pits22575-bib-0010] Bhattacharya, K. (2017). Fundamentals of qualitative research: A practical guide. Taylor & Francis.

[pits22575-bib-0011] Braun, V. , & Clarke, V. (2006). Using thematic analysis in psychology. Qualitative Research in Psychology, 3(2), 77–101. 10.1191/1478088706qp063oa

[pits22575-bib-0012] Catalano, R. F. , Berglund, M. L. , Ryan, J. A. M. , Lonczak, H. S. , & Hawkins, J. D. (2002). Positive youth development in the United States: Research findings on evaluations of positive youth development programs. Prevention & Treatment, 591(1), 98–124. 10.1037/1522-3736.5.1.515a

[pits22575-bib-0013] Charmaz, K. (2006). Constructing grounded theory: A practical guide through qualitative analysis. Sage Publications.

[pits22575-bib-0014] Collie, R. J. , Shapka, J. D. , & Perry, N. E. (2011). Predicting teacher commitment: The impact of school climate and social‐emotional learning. Psychology in the Schools, 48(10), 1034–1048. 10.1002/pits.20611

[pits22575-bib-0015] Crenshaw, K. (1991). Mapping the margins: Intersectionality, identity politics, and violence against women of color. Stanford Law Review, 43(6), 1241–1299. 10.2307/1229039

[pits22575-bib-0016] Creswell, J. W. , & Poth, C. N. (2016). Qualitative inquiry and research design: Choosing among five approaches. Sage Publications.

[pits22575-bib-0017] Debnam, K. , Johnson, S. , Waasdorp, T. , & Bradshaw, C. (2014). Equity, connection, and engagement in the school context to promote positive youth development. Journal of Research on Adolescence, 24(3), 447–459. 10.1111/jora.12083

[pits22575-bib-0018] Dixson, A. D. , Rousseau, C. K. , Anderson, C. R. & Donnor, J. K. (Eds.). (2006). Critical race theory in education: All God's children got a song. Taylor & Francis.

[pits22575-bib-0062] Dmitrieva, J. , Monahan, K. C. , Cauffman, E. , & Steinberg, L. (2012). Arrested development: The effects of incarceration on the development of psychosocial maturity. Development and Psychopathology, 24(3), 1073–1090. 10.1017/S0954579412000545 22781872

[pits22575-bib-0019] Dumas, M. J. (2016). Against the dark: Antiblackness in education policy and discourse. Theory into Practice, 55(1), 11–19. 10.1080/00405841.2016.1116852

[pits22575-bib-0020] Durlak, J. A. , Domitrovich, C. E. , Weissberg, R. P. , & Gullotta, T. P. (2015). Handbook of social and emotional learning: Research and practice. The Guilford Press.

[pits22575-bib-0021] Durlak, J. A. , Weissberg, R. P. , Dymnicki, A. B. , Taylor, R. D. , & Schellinger, K. B. (2011). The impact of enhancing students' social and emotional learning: A meta‐analysis of school‐based universal interventions. Child Development, 82(1), 405–432. 10.1111/j.1467-8624.2010.01564.x 21291449

[pits22575-bib-0022] Dutil, S. (2020). Dismantling the school‐to‐prison pipeline: A trauma‐informed, critical race perspective on school discipline. Children & Schools, 42(3), 171–178. 10.1093/cs/cdaa016

[pits22575-bib-0023] Espelage, D. L. , Low, S. , Polanin, J. R. , & Brown, E. C. (2013). The impact of a middle school program to reduce aggression, victimization, and sexual violence. Journal of Adolescent Health, 53(2), 180–186. 10.1016/j.jadohealth.2013.02.021 23643338

[pits22575-bib-0063] Espinoza, O. (2007). Solving the equity-equality conceptual dilemma: A new model for analysis of the educational process. Educational Research (Windsor), 49(4), 343–363. 10.1080/00131880701717198

[pits22575-bib-0024] Fish, J. M. (Ed.). (2001). Race and intelligence: Separating science from myth. L. Erlbaum.

[pits22575-bib-0025] Forero, R. , Nahidi, S. , De Costa, J. , Mohsin, M. , Fitzgerald, G. , Gibson, N. , McCarthy, S. , & Aboagye‐Sarfo, P. (2018). Application of four‐dimension criteria to assess rigour of qualitative research in emergency medicine. BMC Health Services Research, 18(1), 120. 10.1186/s12913-018-2915-2 29454350 PMC5816375

[pits22575-bib-0026] Gatti, U. , Tremblay, R. E. , & Vitaro, F. (2009). Iatrogenic effect of juvenile justice. Journal of Child Psychology and Psychiatry, 50(8), 991–998. 10.1111/j.1469-7610.2008.02057.x 19673053

[pits22575-bib-0027] Giddings, G. J. (2001). Infusion of Afrocentric content into the school curriculum: Toward an effective movement. Journal of Black Studies, 31(4), 462–482. 10.1177/002193470103100405

[pits22575-bib-0028] Ginwright, S. (2018). The future of healing: Shifting from trauma informed care to healing centered engagement. Occasional, 25.

[pits22575-bib-0029] Grace, J. E. , & Nelson, S. L. (2019). “Tryin' to Survive”: Black male students' understandings of the role of race and racism in the school‐to‐prison pipeline. Leadership and Policy in Schools, 18(4), pp. 664–680. 10.1080/15700763.2018.1513154

[pits22575-bib-0030] Gregory, A. , & Fergus, E. (2017). Social and emotional learning and equity in school discipline. The Future of Children, 27(1), 117–136. 10.1353/foc.2017.0006

[pits22575-bib-0031] Herrenkohl, T. I. , Jones, T. M. , Lea, C. H. , & Malorni, A. (2020). Leading with data: Using an impact‐driven research consortium model for the advancement of social emotional learning in schools. American Journal of Orthopsychiatry, 90(2), 283–287. 10.1037/ort0000435 31789545

[pits22575-bib-0032] Hoffman, D. M. (2009). Reflecting on social emotional learning: A critical perspective on trends in the United States. Review of Educational Research, 79(2), 533–556. 10.3102/0034654308325184

[pits22575-bib-0033] Jagers, R. J. , Rivas‐Drake, D. , & Williams, B. (2019). Transformative social and emotional learning (SEL): Toward SEL in service of educational equity and excellence. Educational Psychologist, 54(3), 162–184. 10.1080/00461520.2019.1623032

[pits22575-bib-0034] Jernigan, M. M. , & Daniel, J. H. (2011). Racial trauma in the lives of black children and adolescents: Challenges and clinical implications. Journal of Child & Adolescent Trauma, 4(2), 123–141. 10.1080/19361521.2011.574678

[pits22575-bib-0035] Kayler‐Jones, C. (2019, May). *When SEL is used as another form of policing. Communities for Just Schools*. Medium. https://medium.com/@justschools/when-sel-is-used-as-another-form-of-policing-fa53cf85dce4

[pits22575-bib-0036] Kozol, J. (2005). The shame of the nation: The restoration of apartheid schooling in America. Broadway Books.

[pits22575-bib-0037] Ladson‐Billings, G. (1995). Toward a theory of culturally relevant pedagogy. American Educational Research Journal, 32(3), 465–491. 10.2307/1163320

[pits22575-bib-0038] Lock, A. , & Strong, T. (2010). Social constructionism: Sources and stirrings in theory and practice. Cambridge University Press.

[pits22575-bib-0039] Merriam, S. B. (2009). Qualitative research: A guide to design and implementation. Jossey‐Bass.

[pits22575-bib-0040] Nadal, K. L. , Griffin, K. E. , Wong, Y. , Hamit, S. , & Rasmus, M. (2014). The impact of racial microaggressions on mental health: Counseling implications for clients of color. Journal of Counseling and Development, 92(1), 57–66. 10.1002/j.1556-6676.2014.00130.x

[pits22575-bib-0041] Noguera, P. A. (2009). The trouble with black boys:… And other reflections on race, equity, and the future of public education. John Wiley & Sons.

[pits22575-bib-0042] Ortega, L. , Lyubansky, M. , Nettles, S. , & Espelage, D. L. (2016). Outcomes of a restorative circles program in a high school setting. Psychology of Violence, 6(3), 459–468. 10.1037/vio0000048

[pits22575-bib-0043] Paris, D. , & Alim, H. S. (2014). What are we seeking to sustain through culturally sustaining pedagogy? A loving critique forward. Harvard Educational Review, 84(1), 85–100. 10.17763/haer.84.1.982l873k2ht16m77

[pits22575-bib-0044] Parson, L. (2019). Considering positionality: The ethics of conducting research with marginalized groups. In L. Atkins & V. Duckworth (Eds.). Research methods for social justice and equity in education (pp. 15–32). Palgrave Macmillan.

[pits22575-bib-0045] Reskin, B. F. (2000). Getting it right: Sex and race inequality in work organizations. Annual Review of Sociology, 26(1), 707–709. 10.1146/annurev.soc.26.1.707

[pits22575-bib-0046] Rolón‐Dow, R. , & Davison, A. (2020). Theorizing racial microaffirmations: A critical race/latcrit approach. Race Ethnicity and Education, 24(2), 245–261. 10.1080/13613324.2020.1798381

[pits22575-bib-0047] Rowe, H. L. , & Trickett, E. J. (2018). Student diversity representation and reporting in universal school‐based social and emotional learning programs: Implications for generalizability. Educational Psychology Review, 30(2), 559–583. 10.1007/s10648-017-9425-3

[pits22575-bib-0048] Rowe, M. (2008). Micro‐affirmations and micro‐inequities. Journal of the International Ombudsman Association, 1(1), 45–48.

[pits22575-bib-0049] Sellman, E. , Cremin, H. , & McCluskey, G. (2013). Restorative approaches to conflict in schools: Interdisciplinary perspectives on whole school approaches to managing relationships. Routledge.

[pits22575-bib-0050] Simmons, D. (2019). Why we can't afford to whitewashed social and emotional learning. Association of Supervision and Curriculum Development, ASCD. Education Update 61(4).​ http://www.ascd.org/publications/newsletters/education_update/apr19/vol61/num04/Why_We_Can%27t_Afford_Whitewashed_Social-Emotional_Learning.aspx

[pits22575-bib-0051] Skiba, R. , Ormiston, H. , Martinez, S. , & Cummings, J. (2016). Teaching the social curriculum: Classroom management as behavioral instruction. Theory into Practice, 55(2), 120–128. 10.1080/00405841.2016.1148990

[pits22575-bib-0052] Skiba, R. J. , Horner, R. H. , Chung, C.‐G. , Rausch, M. K. , May, S. L. , & Tobin, T. (2011). Race is not neutral: A national investigation of African American and Latino disproportionality in school discipline. School psychology review, 40(1), 85–107. 10.1080/02796015.2011.12087730

[pits22575-bib-0053] Stich, A. E. , Cipollone, K. , Nikischer, A. , & Weis, L. (2012). Walking the methodological tightrope: Researcher dilemmas inside an urban school district in times of public disinvestment. Qualitative Inquiry, 18(6), 463–474. 10.1177/1077800412442815

[pits22575-bib-0054] Taylor, K. Y. (2016). From #BlackLivesMatter to black liberation. Haymarket Books.

[pits22575-bib-0055] Taylor, R. D. , Oberle, E. , Durlak, J. A. , & Weissberg, R. P. (2017). Promoting positive youth development through school‐based social and emotional learning interventions: A meta‐analysis of follow‐up effects. Child Development, 88(4), 1156–1171. 10.1111/cdev.12864 28685826

[pits22575-bib-0056] The Aspen Institute: Education and Society Program . (2018). *Pursuing social and emotional development through a racial equity lens: A call to action*. https://assets.aspeninstitute.org/content/uploads/2018/05/Aspen-Institute_Framing-Doc_Call-to-Action.pdf?_ga=2.72309142.42349713.1603470735-878698989.1603470735

[pits22575-bib-0057] Thompson, E. (2010). Addressing institutional structural barriers to student achievement. *Race* . Gender & Class, 17(1/2), 51–57.

[pits22575-bib-0058] Topor, A. , Bøe, T. D. , & Larsen, I. B. (2018). Small things, micro‐affirmations and helpful professionals everyday recovery‐orientated practices according to persons with mental health problems. Community Mental Health Journal, 54(8), 1212–1220. 10.1007/s10597-018-0245-9 29423684 PMC6208994

[pits22575-bib-0059] Wang, S. , Rubie‐Davies, C. M. , & Meissel, K. (2018). A systematic review of the teacher expectation literature over the past 30 years. Educational Research and Evaluation, 24(3‐5), 124–179. 10.1080/13803611.2018.1548798

[pits22575-bib-0060] Whitney, D. G. , & Peterson, M. D. (2019). US national and state‐level prevalence of mental health disorders and disparities of mental health care use in children. JAMA Pediatrics, 173(4), 389–391. 10.1001/jamapediatrics.2018.5399 30742204 PMC6450272

